# Examination of the impact of curiosity and fatigue on brain condition

**DOI:** 10.1038/s41598-025-97796-5

**Published:** 2025-04-15

**Authors:** Yan Ni, Keisuke Kokubun, Kiyotaka Nemoto, Yoshinori Yamakawa

**Affiliations:** 1 Graduate School of Economics, Kyoto University, Kyoto, Japan; 2 Open Innovation Institute, Kyoto University, Kyoto, Japan; 3 Graduate School of Management, Kyoto University, Kyoto, Japan; 4 Department of Psychiatry, Institute of Medicine, University of Tsukuba, Tsukuba, Japan; 5 Institute of Innovative Research, Tokyo Institute of Technology, Meguro, Tokyo Japan; 6 ImPACT Program of Council for Science, Technology and Innovation (Cabinet Office, Government of Japan), Chiyoda, Tokyo Japan; 7 Office for Academic and Industrial Innovation, Kobe University, Kobe, Japan; 8 Brain Impact, Kyoto, Japan

**Keywords:** Neuroscience, Cognitive neuroscience, Neurophysiology

## Abstract

With a growing emphasis on cognitive well-being, this study investigates factors influencing brain health, measured by Fractional Anisotropy Brain Healthcare Quotient (FA-BHQ). We find a positive correlation between curiosity, assessed by the Curiosity and Exploration Inventory-II (CEI-II), and brain health as indicated by FA-BHQ. Conversely, fatigue, evaluated using the Chalder Fatigue Scale (CFS), demonstrates a negative correlation with brain health. Furthermore, curiosity and fatigue serve as significant mediators in the relationship between these factors and FA-BHQ, respectively. Specific brain regions, including the corpus callosum, internal capsule, fornix, and posterior thalamic radiation, show significant negative correlations with fatigue, while the corpus callosum, fornix, internal capsule, corona radiata, external capsule, cingulum, and posterior thalamic radiation exhibit significant positive correlations with curiosity. Notably, this study highlights that more brain regions exhibit significant correlations with curiosity compared to fatigue, shedding light on the growing recognition of curiosity’s role in influencing brain health and emphasizing the importance of considering curiosity in the research on cognitive well-being.

## Introduction

Curiosity, as delineated by Loewenstein in 1994^1^, is a fundamental element of cognitive processes, influencing knowledge acquisition, decision-making, and playing a significant role in one’s overall development. Conversely, fatigue can be perceived as an illness, the symptoms of fatigue can be separated into physical and mental. Mental fatigue is often linked psychobiological state of exhaustion that results from extended engagement in cognitively demanding tasks, leading to diminished cognitive performance^2,3^. While research on the relationship between curiosity and brain health remains limited, several studies have explored the neural mechanisms underlying curiosity driven by novelty and unpredictability^4^. Past literature suggests some overlap in brain regions involved in these processes, indicating that regions associated with reward and emotion may play a role in curiosity^5^. On the other hand, previous research has extensively examined the impact of physical fatigue on brain activities. For example, Tran et al. found that monitoring and addressing fatigue involves changes in theta activity at various brain sites, serving as a reliable biomarker for mental exhaustion. Additionally, Tedeschi et al. showed that high levels of fatigue are associated with increased abnormal white matter fraction (AWM-f) and reduced white matter fraction (WM-f) and gray matter fraction (GM-f). A high score on the Fatigue Severity Scale (FSS) was significantly linked to decreased WM-f and GM-f. However, the research on the intricate relationship between fatigue and curiosity is still unexplored at this stage.

In this research, we employ Fractional Anisotropy Brain Healthcare Quotient (FA-BHQ) as a tool to measure brain health, building on the work of Nemoto et al. 2007^6^ FA-BHQ is utilized to quantify brain health and explore its relationship with curiosity and fatigue. Numerous previous studies have explored the intricate relationship between fatigue and fractional anisotropy (FA), consistently revealing a negative association between them. For instance, Wilting et al.^7^ noted reduced thalamic fractional anisotropy in patients with cognitive fatigue exhibited reduced fractional anisotropy (FA) and increased mean diffusivity (MD) in the thalamus compared to both non-fatigued MS patients and healthy controls., Wu, Inman et al.^8^ also found that higher fatigue scores on clinical assessment of ankylosing spondylitis (AS) were associated with lower fractional anisotropy values on inferior fronto-occipital fasciculi, superior/inferior longitudinal fasciculi, and corticothalamic tracts. Yarraguntla, et al.^9^ reported a significant negative correlation between the pallidal volume and fractional anisotropy of the right temporal cortex and FSS scores. Genova, Rajagopalan et al.^10^ uses functional MRI (fMRI) and diffusion tensor imaging (DTI) to examine brain activation and white matter integrity and observed diminished FA in the anterior internal capsule in individuals with higher self-reported fatigue on the FSS which assesses “trait” fatigue in MS group. Conversely, the research regarding the relationship between curiosity and fractional anisotropy is relatively limited. Valji, et al.^11^ investigated curiosity levels using Epistemic Curiosity (EC) scales and found a notable positive correlation between FA in the entire fornix and interest EC. Importantly, a balancing effect of curiosity and fatigue has been observed in a previous study conducted by Garrosa et al.^12^, revealing that curiosity reduces exhaustion and promotes daily engagement among college students.

In light of this context, we hypothesize that: (1) brain health, as measured by FA-BHQ, is positively correlated with curiosity; (2) FA-BHQ is negatively correlated with fatigue; (3) fatigue mediates the relationship between curiosity and FA; (4) curiosity mediates the relationship between fatigue and FA.

## Materials and methods

### Subjects

From October 2016 to February 2023, a total of 578 healthy participants (231 women, 347 men), were recruited in Kyoto, Kobe and Tokyo, Japan.

**Fig. 1:**
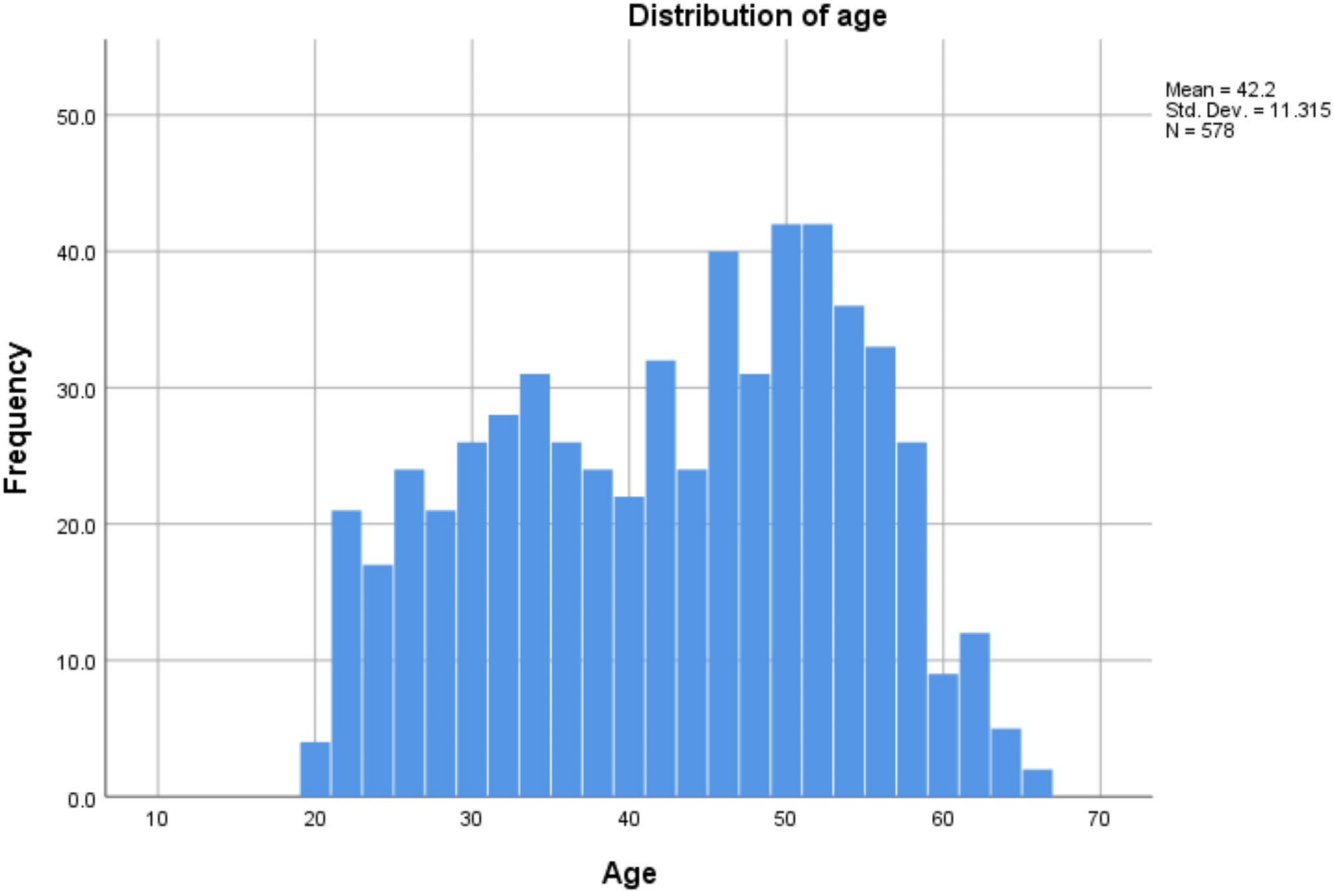
The distribution of the age of the participants.

Participants are employed persons gathered by member companies of the BHQ Consortium, which is a study group aimed at maintaining and improving the brain health of individuals, or by a personal service company. According to the self-reports, none of the recruited participants had records of neurological, psychiatric, or other medical conditions that could affect the central nervous system. This study was approved by all four academic institutions, namely Ethics Committee of Kyoto University (Approval Number 27-P-13), Tokyo Institute of Technology (Approval Number A16038), the University of Tokyo (Approval Number 402-2) and RIKEN Center for Life Science Technologies (Approval Number KOBE-IRB-15-13). The research was conducted following the institutes’ guidelines and regulations. All participants provided written informed consent before participation, and their anonymity was maintained. Figure 1 shows a histogram showing the distribution of age.

### Curiosity scales

In our study, we utilized the Japanese version of the Curiosity and Exploration Inventory-II (CEI-II), to evaluate individuals’ curiosity levels. CEI-II was originally developed by Kashdan et al. in 2009^13^, and the Japanese version was tested by Nishikawa et al. in 2015^14^ and its effectiveness was confirmed. The questionnaire consists of ten items, and participants were asked to rate their agreement with each item on a 5-point Likert scale, ranging from ‘1’ (Not applicable at all) to ‘5’ (Very applicable). These questions encompass various traits related to curiosity, such as the inclination to seek out novel experiences, embrace uncertainty, and engage in complex challenges.

Our decision to employ the CEI-II in this study because this survey suits our research focus. Firstly, the inclination of CEI-II fits with the theme of our research. In Litman, 2006 and Litman, 2019^15,16^, they established the concept of Interest and Deprivation curiosity. They have motivational differences driving curiosity. I-Type curiosity is associated with enjoyment, D-Type curiosity stems from an urgent need to resolve informational gaps. In this paper, we aimed to assess the positive side of curiosity and CEI-II studies suit the focus. Secondly, the CEI-II is widely recognized as one of the most commonly used measures of curiosity internationally. In the research conducted by Valji et al.^11^, they found that differences in distinct dimensions of curiosity systematically map onto specific white matter tracts that underlie well-characterized brain networks.

### Fatigue

In our research, we have utilized the Chalder Fatigue Scale (CFS) consisting of 11 items. The CFS is a comprehensive instrument designed to assess the fatigue experienced by individuals, encompassing both physical and mental aspects. It was initially developed by Chalder et al. in 1993^17^ and later revised by Cella et al. in 2010^18^ to include 11 items. Responses to the first ten questions are scored on a 4-point scale, ranging from ‘1’ (indicating ‘No’) to ‘4’ (signifying ‘A great deal’). Question 11 assesses memory and employs a different scale: ‘1’ (Better than usual), ‘2’ (Same as usual), ‘3’ (Worse than usual), and ‘4’ (Much worse than usual).

Our decision to employ the sum of scores for each method in our study was made to facilitate a thorough exploration of the relationship between fatigue and specific facets of physical and mental well-being. The use of the CFS comprehensively accesses fatigue and is supported by its widespread use in the medical research field. Notably, Uehara et al. conducted research on the relationship between fatigue and periodontitis, utilizing the CFS to measure patient fatigue levels. Furthermore, given previous literature demonstrating negative correlations between FA values and fatigue levels, we believe that adopting the CFS is a suitable choice for our study^9,10,19^.

**Table 1:** Reliability statistics of the internal consistency analysis.

	Curiosity	Fatigue
Cronbach’s Alpha	0.902	0.920
No. of Items	10	11
No. of Cases	578	578

### Internal consistency analysis

To access the two sets of questionnaires, namely the CEI-II comprising 10 items and the CFS with 11 items, a reliability analysis was conducted to assess the internal consistency of the questionnaire items. The analysis involved measuring Cronbach’s Alpha for both questionnaires and the obtained results are presented in the Table 1. Notably, the Cronbach’s Alpha values for Curiosity and Fatigue are 0.902 and 0.920, respectively. These values, both exceeding 0.9, signify a high level of internal consistency for both variables.

### Demographic scales

In our research, we considered several demographic variables and control factors. These included Body Mass Index (BMI), calculated by dividing an individual’s weight in kilograms by the square of their height in meters; sex, which was represented as a binary variable where “1” indicated males and “2” indicated females; the locations where MRI scans were conducted, and age. The venues for MRI scans were categorized into different binary variables, with “1” denoting the participant received the checkup at the specific venue and “0” indicating otherwise. Participants were gathered at various institutions with questionnaires and brain images collected on the same day.

### MRI data acquisition

All magnetic resonance imaging (MRI) data were procured utilizing a 3-Tesla scanner. A three-dimensional (3D) T1-weighted high-resolution structural image and a Diffusion Tensor Imaging image were acquired in the process. Parameters for each MRI scanner is described in Table 2.

**Table 2:** Summary of MRI scanners and pulse sequences used in 4 experiment venues.

		vENUE 1	VENUE 2	VENUE 3	VENUE 4_1	VENUE 4_2
	Experiment Venue	Kyoto	Tokyo	RIKEN	TITech	TITech
	Manufacturer	SIEMENS	SIEMENS	SIEMENS	GE	SIEMENS
	Model Name	Verio	Prisma	Prisma	Signa HDxt	Prisma
	Magnetic Field Strength	3T				
3DT1WI	Sequence	MPRAGE	MPRAGE	MPRAGE	FSPGR	MPRAGE
	Echo Time (ms)	2.5	2.5	2.2	2.8	2.5
	Repetition Time (ms)	1900	1900	2400	7.0	1900
	Flip Angle (degree)	9	9	8	11	9
	Acquisition Matrix	256 × 256	256 × 240	256 × 256	256 × 256	256 × 256
	Pixel Spacing (mm)	1.0	1.0	1.0	1.0	1.0
	Slice Thickness (mm)	1.0	1.0	1.0	1.2	1.2
DTI	Sequence	Diffusion-weighted spin-echo EPI				
	Echo Time (ms)	81	81	82	72	81
	Repetition Time (ms)	14,100	14,100	3700	16,000	9000
	Flip Angle (degree)	90	90	90	90	90
	Acquisition Matrix	114 × 114	114 × 114	118 × 118	128 × 128	114 × 114
	Pixel Spacing (mm)	2.0	2.0	1.7	1.0	2.0
	Slice Thickness (mm)	2	2	1.7	2.5	2
	Non-collinear gradient directions	30	30	30	30	30
	b-values	0/1000	0/1000	0/1000	0/1000	0/1000

3DT1WI: three-dimensional T1-weighted imaging; DTI: Diffusion Tensor Imaging; MPRAGE: Magnetization Prepared - Rapid Gradient Echo; FSPGR: Fast Spoiled Gradient Recalled; EPI: Echo-Planar Imaging.

### GM-BHQ and FA-BHQ

The preprocessing of T1-weighted images involved several stages by using Statistical Parametric Mapping 12 (SPM12) in MATLAB R2021b. First, each 3D-T1 image was segmented into gray matter (GM), white matter (WM), and cerebrospinal fluid (CSF) images. The GM images were then subjected to normalization using an exponentiated lie algebra (DARTEL) algorithm, which included a modulation step for preserving regional volume. Subsequently, all normalized images were smoothed with an 8 mm Gaussian kernel. Intracranial volume (ICV) was determined by summing the GM, WM, and CSF images for each subject. To address differences in whole-brain volume among participants, proportional GM images were generated, resulting in mean and standard deviation (SD) images. The GM -BHQ, akin to an intelligence quotient (IQ), was calculated with a mean of BHQ 100 and an SD of 15 BHQ points. According to this definition, approximately 68% of the population falls within BHQ 85 to BHQ 115, and 95% within BHQ 70 to BHQ 130. Individual GM quotient images were computed using the formula: 100 + 15 * (individual proportional GM - mean) / SD. Finally, the Automatic Anatomical Labeling (AAL) atlas was utilized to extract regional GM quotients and create participant-specific GM-BHQs.

The DTI data were preprocessed using FMRIB Software Library (FSL) version 6.0.5. First, all diffusion images were aligned with the initial b0 image, and eddy correction was applied for motion and eddy current distortion corrections. Next, we used DTIFit to compute fractional anisotropy (FA) images, and these FA images were spatially normalized to the standard Montreal Neurological Laboratory (MNI) space using FLIRT and FNIRT. Following the standardized Brain Healthcare Quotient (BHQ) protocol established by Nemoto et al. 2017^6^, we maintained consistent preprocessing parameters to ensure comparability with existing BHQ research. Mean and standard deviation (SD) images were created from all FA images, and individual FA quotient images were calculated using the formula: 100 + 15 * (individual FA - mean) / SD. Regional FA quotients were obtained using the Johns Hopkins University (JHU) DTI-based white matter atlas and were averaged across regions to generate participant-specific FA-BHQ scores. The selection of the JHU atlas for white matter and AAL atlas for gray matter analyses was based both on their established reliability and their permissive licensing terms, facilitating broader research reproducibility. Nemoto et al. 2017^6^ provides more information. In our previous studies, we discovered that whole-brain GM-BHQ exhibited positive correlations with dietary balance^20^, curiosity^21^, behavioral activation^22^, while showing negative correlations with fatigue^23^ and unhealthy lifestyles^24^. On the other hand, we observed that whole-brain FA-BHQ was positively associated with cognitive function^25,26^, fish diet intake^27^, housing quality^28^, anxiety^25,29^, the personal trait of spiritual growth^25^, training and work engagement^26^, and happiness^29^.

Notably, prior research has established that the Gray Matter Brain Healthcare Quotient (GM-BHQ) is associated with relatively stable and holistic factors, such as socioeconomic status and physical condition. In contrast, the FA-BHQ is linked to relatively short-term and variable psychological factors. To accurately assess the correlations between FA, curiosity, and fatigue, it is essential to account for the physical characteristics of the participants. Therefore, we included BMI, sex, and GM-BHQ as control variables. Additionally, considering previous literature demonstrating a negative relationship between age and FA, we incorporated age as a control variable in our analysis, where FA was the dependent variable. This comprehensive approach aligns with our research objectives, as it ensures the careful examination of the relationships between these variables while controlling for important demographic and physical factors.

To study the relationship between curiosity and fatigue with specific regions of brain, on top of using GM-BHQ and FA-BHQ which focus more on the whole brain, we narrowed the scope into 10 sub-regions of FA-BHQ from the perspective of the region of interest (ROI). This approach is based on the past literature which indicated a structural connectivity in these regions and behavioral functions. In terms of fatigue, Román et al., 2022^30^ has investigated the relationship between the rate of cognitive fatigue and the microstructure of white matter and the basal ganglia in individuals with MS, utilizing advanced diffusion imaging techniques. They found that the compromised white matter along tracts that are associated with fatigue networks, which consists of six regions: the corpus callosum (CC), superior corona radiata (SCR), superior longitudinal fasciculus (SLF), external capsule (EC), internal capsule (IC), and posterior thalamic radiation (PTR), provide evidence that these regions may also be involved in the experience of fatigue. The sagittal stratum has been strongly correlated with language, visual information processing, and cognitive function^31^. In related research, Cockshell and Mathias, 2010^32^ demonstrated an independent causal relationship between subjective fatigue and the rate of cognitive decline. Given these findings, the sagittal stratum was identified as a key ROI in this study. The fornix^33^, cingulum^34^, and uncinate fasciculus^35^ have been identified as critical regions involved in emotional regulation. Previous research has also demonstrated that engaging in emotional regulation tasks can result in mental fatigue, leading to deteriorated performance over time^36^. Consequently, these three regions of interest (ROIs) were included in the scope of our research.

### Statistical analysis

To explore the interrelationships among Curiosity, Fatigue, age, GM-BHQ, FA-BHQ, sex, venue, and BMI, path analysis was employed in alignment with the hypotheses suggesting a positive correlation between FA-BHQ and Curiosity, a negative correlation between FA-BHQ and fatigue, and the mediating roles of Fatigue and Curiosity in the relationship between each other and FA-BHQ. The significance level was set at *p* < 0.05, and the statistical analyses were conducted using SPSS/AMOS version 26 (IBM Corporation, Armonk, NY, USA).

## Results

**Fig. 2:**
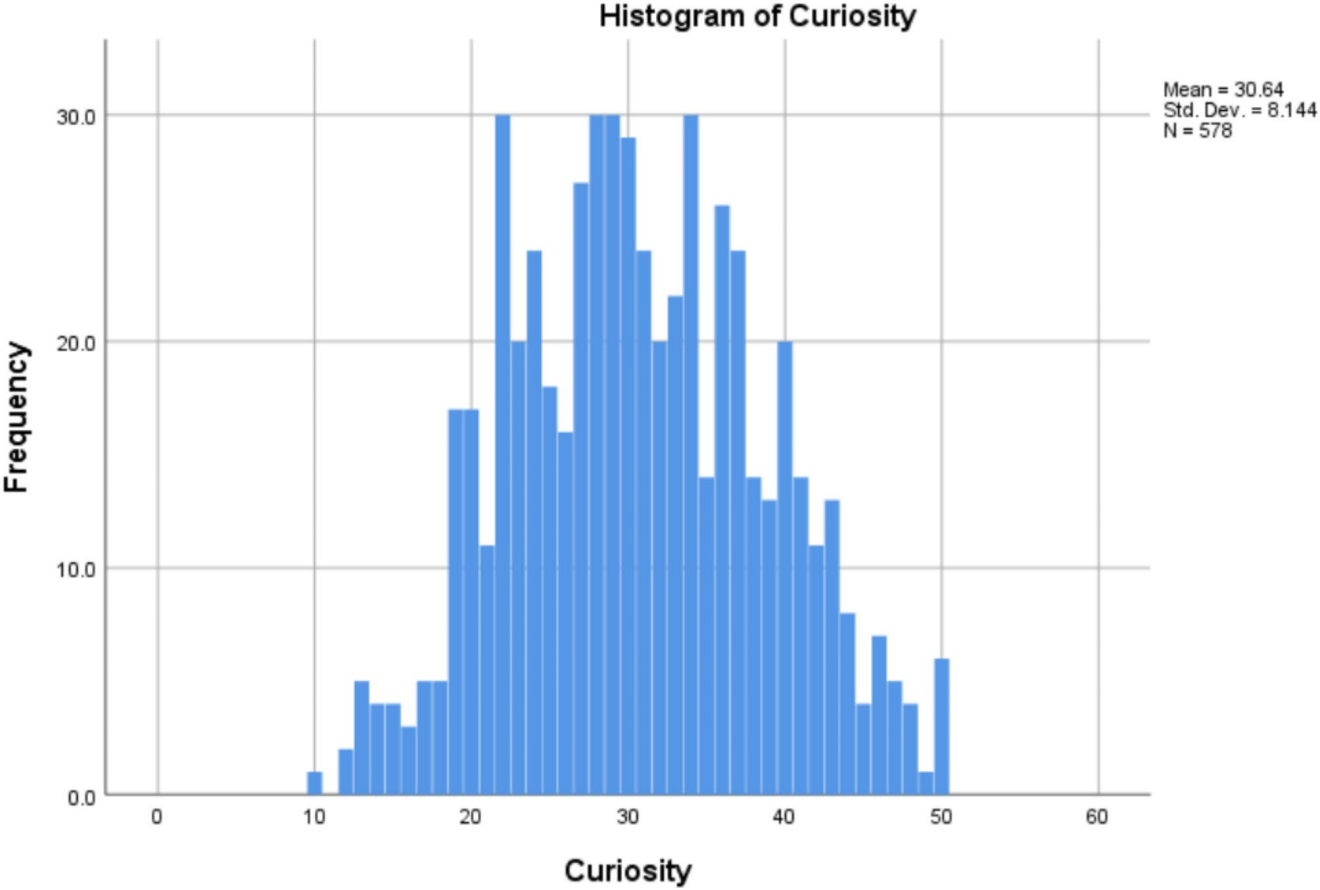
The histogram of Curiosity of the participants.

**Fig. 3:**
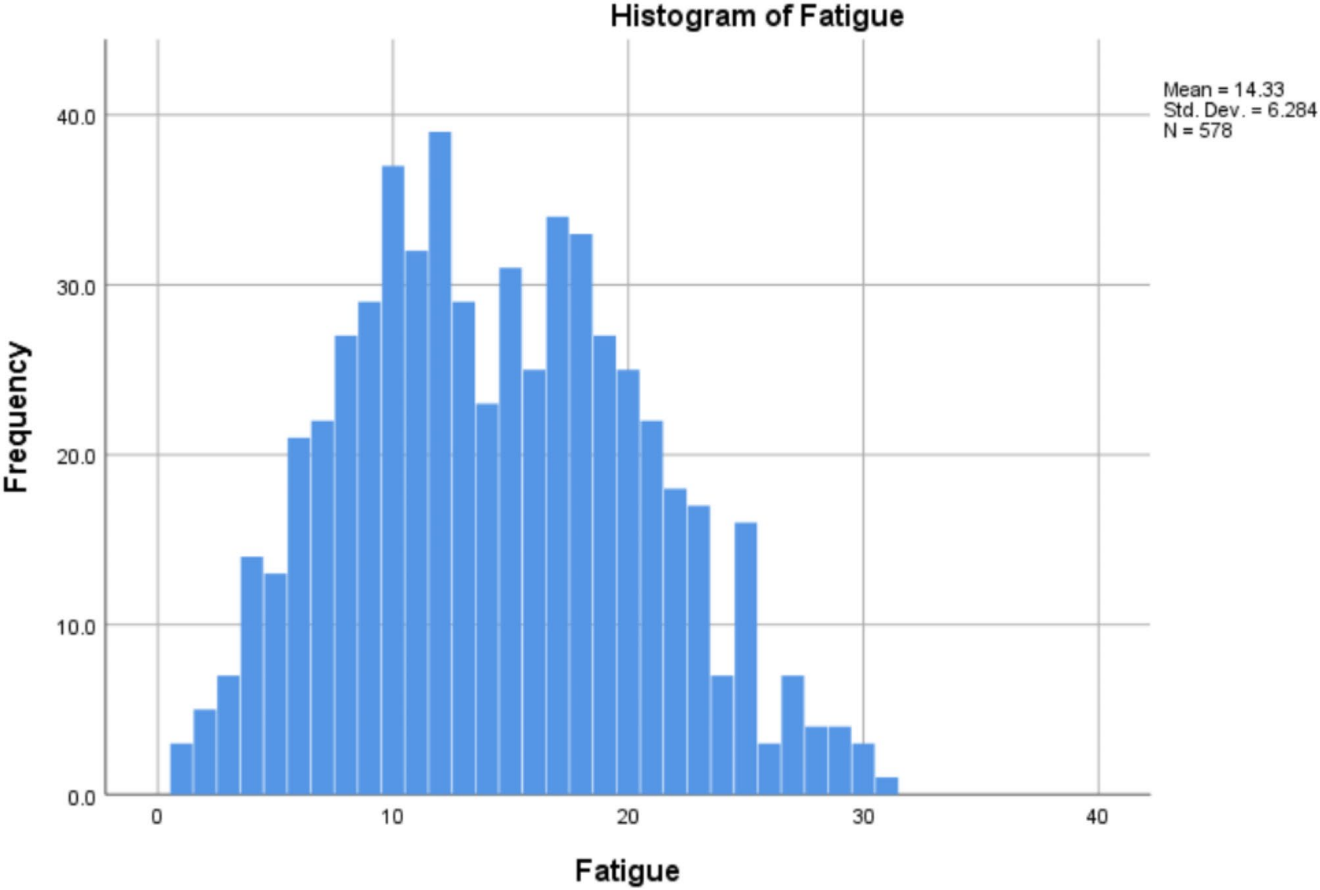
The histogram of Fatigue of the participants.

Figures 2 and 3 are the histograms showing the distribution of Curiosity and Fatigue scores.

Given that the distribution violates the assumption of ANOVA on equal variance, we conducted the Kruskal-Wallis Test and Chi-square Test to assess the independence of continuous and categorical independent variables across various experiment venues. The results, as shown in Table 3, indicate a statistically significant difference among experiment venues for all independent variables, including Curiosity (*H* = 77.19, *p* < 0.001), Fatigue (*H* = 46.74, *p* < 0.001), age (*H* = 28.22, *p* < 0.001), BMI (*H* = 40.17, *p* < 0.001), GM-BHQ (*H* = 40.54, *p* < 0.001), FA-BHQ (*H* = 65.16, *p* < 0.001), and Sex (χ2 = 60.37, *p* < 0.001). Consequently, we opted to employ the entire sample in a unified model, while also controlling for the venue of the experiment in subsequent analyses.

**Table 3:** Independence test result of variables in different venues.

	Venue 1		Venue 2		Venue 3		Venue 4_1		Venue 4_2		H	*p*-value
	Mean	SD	Mean	SD	Mean	SD	Mean	SD	Mean	SD		
Curiosity	25.72	7.38	24.89	7.04	27.97	7.95	28.28	4.89	32.50	7.85	77.19	***
Fatigue	12.71	4.85	12.86	5.11	8.24	3.86	13.93	5.04	15.23	6.52	46.74	***
Age	38.74	17.263	48.25	10.99	41.59	10.99	35.55	4.79	42.37	10.75	28.22	***
BMI	22.12	2.61	21.78	2.81	22.14	2.40	20.17	1.41	23.23	3.74	40.17	***
GM-BHQ	102.93	11.13	99.24	6.07	101.79	7.94	110.51	4.55	102.29	8.01	40.54	***
FA-BHQ	97.54	4.66	99.36	2.89	104.20	2.35	99.54	2.32	100.21	3.20	65.16	***
	N	%	N	%	N	%	N	%	N	%	χ2	
Male	43	74.1%	24.00	42.9%	22.00	75.9%	0.00	0.0%	258.00	63.5%	60.37	***
Female	15	25.9%	32.00	57.1%	7.00	24.1%	29.00	100.0%	148.00	36.5%		

*n* = 578; * *p* < 0.05; ** *p* < 0.01; *** *p* < 0.001.

Table 4 provides descriptive statistics for all subjects and bivariate correlation coefficients between pairwise variables. Curiosity displayed correlations with Fatigue (*r* = -0.099, *p* < 0.05), sex (*r* = -0.126, *p* < 0.01), BMI (*r* = 0.095, *p* < 0.05), and FA-BHQ scores (*r* = 0.138, *p* < 0.01). In contrast, Fatigue exhibited correlations with age (*r* = -0.136, *p* < 0.01), FA-BHQ (*r* = − 0.087, *p* < 0.05), sex (*r* = 0.104, *p* < 0.05), and GM-BHQ scores (*r* = 0.108, *p* < 0.01). FA-BHQ demonstrated significant correlations at the 5% level with all variables except sex and BMI. Conversely, GM-BHQ showed a significant correlation at the 5% level with all variables except Curiosity.

**Table 4:** Pairwise partial correlation test results among all the variables.

	Mean	SD	Curiosity	Fatigue	Sex	Age	BMI	GM-BHQ
Curiosity	30.64	8.14						
Fatigue	14.33	6.28	-0.099*					
Sex	1.40	0.49	-0.126**	0.104*				
Age	42.20	11.32	-0.037	-0.136*	-0.155***			
BMI	22.77	3.50	0.095*	0.112**	-0.263***	0.145***		
GM-BHQ	102.45	8.32	-0.038	0.108**	0.434***	-0.727***	-0.308***	
FA-BHQ	100.03	3.50	0.138**	-0.087*	-0.036	-0.324***	-0.079	0.314***

*n* = 578; * *p* < 0.05; ** *p* < 0.01; *** *p* < 0.001.

**Fig. 4:**
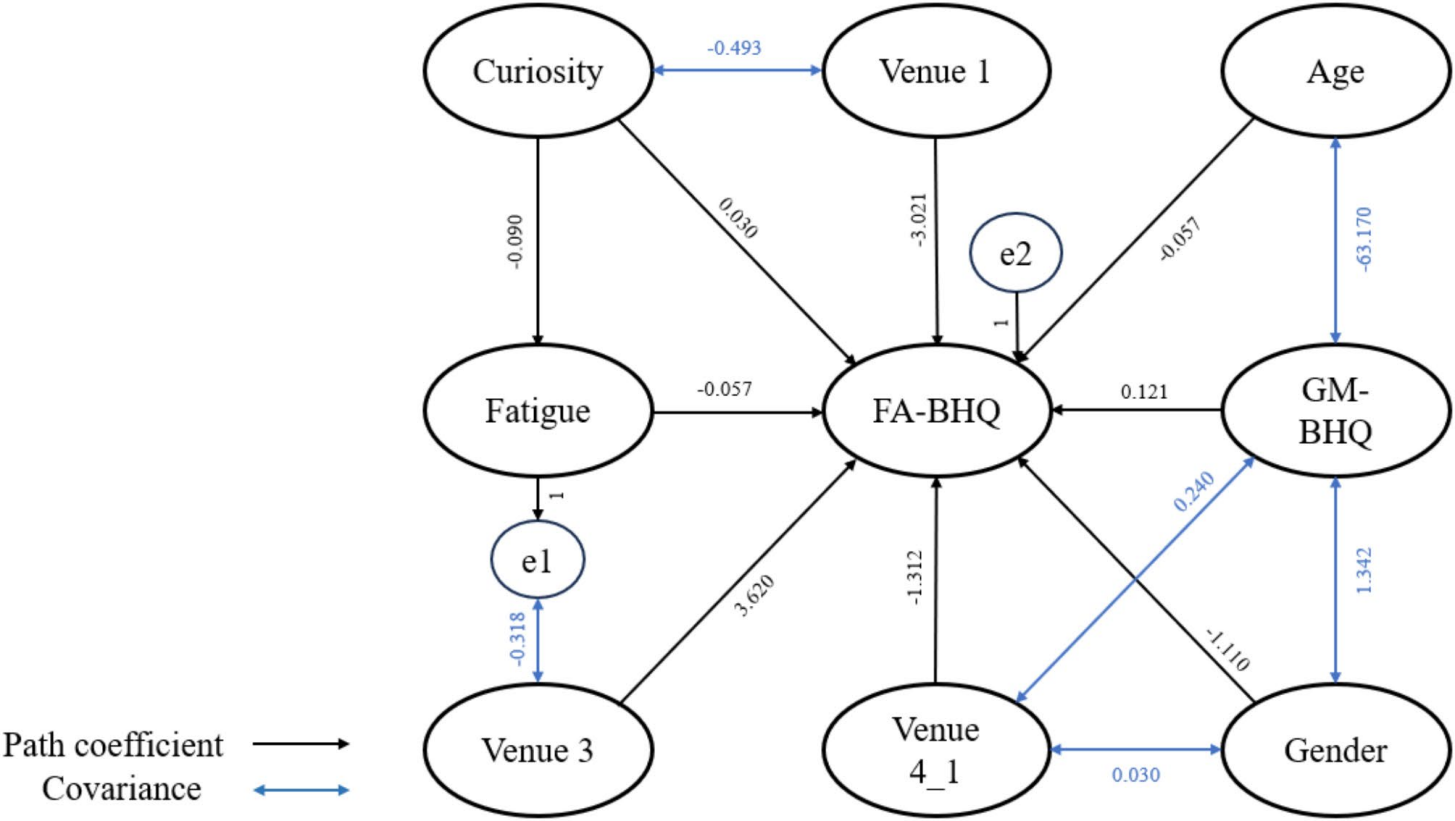
Theorical model 1 linking Curiosity, Fatigue, and FA-BHQ with Fatigue as the mediator between Curiosity and FA-BHQ.

**Fig. 5:**
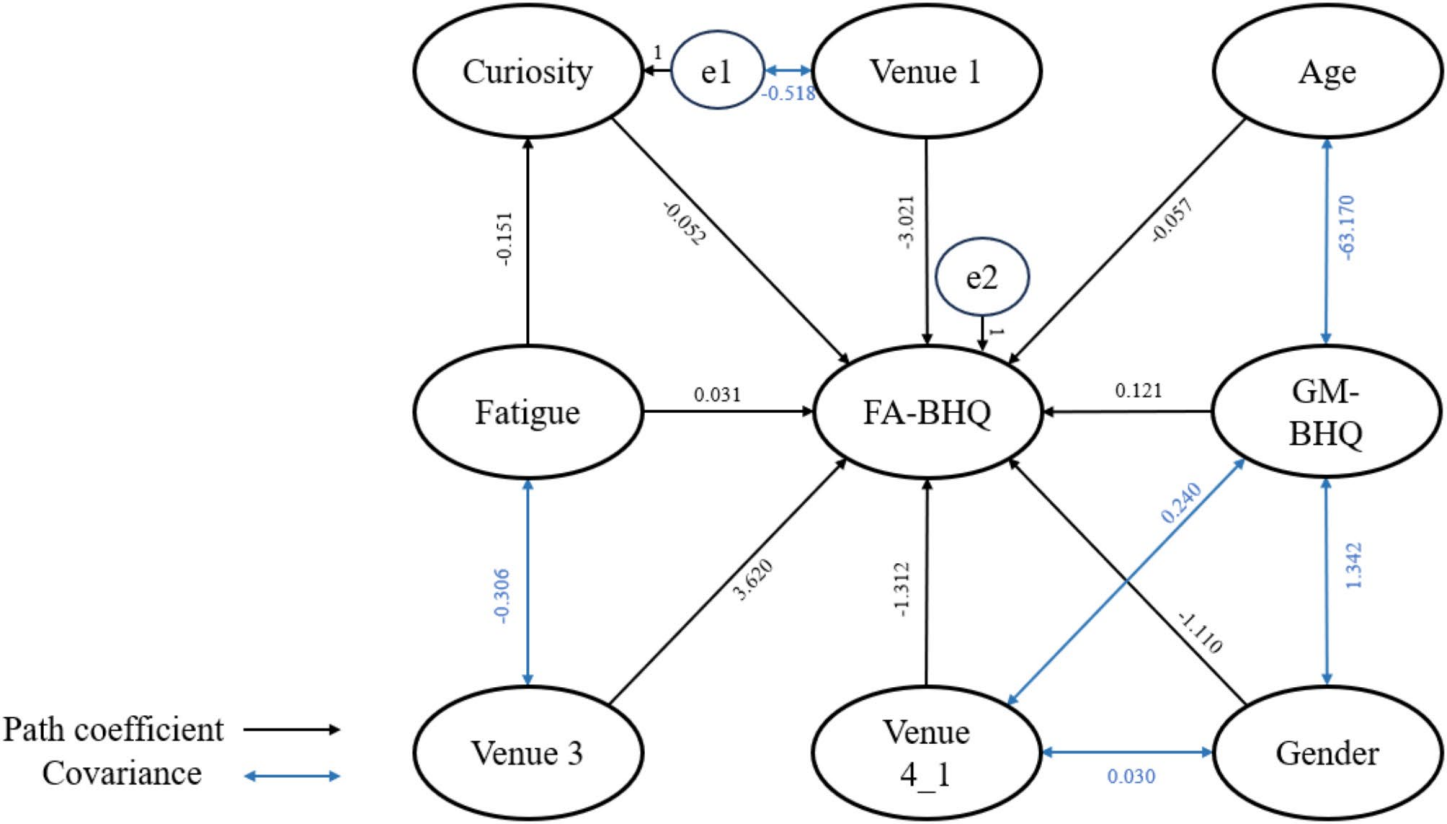
Theorical model 2 linking Curiosity, Fatigue, and FA-BHQ with Curiosity as the mediator between Fatigue and FA-BHQ.

To clarify the connections among Age, BMI, GM-BHQ, FA-BHQ, Curiosity, and Fatigue, we constructed the 2 models illustrated in Figs. 4 and 5, aligning with our proposed hypotheses. Modification indices were employed to refine the model fittings, and standardized path coefficients are presented for reference. The goodness-of-fit indices for the model, indicated in the figure legend, revealed a high level of adaptability. In model 1, fatigue serves as the mediator between curiosity and FA-BHQ, with further control for age, sex, GMBHQ, and the venue for the experiment. In model 2, curiosity takes on the role of the mediator between fatigue and FA-BHQ.

These path diagrams illustrate the observed relationships among Curiosity, Fatigue, and FA-BHQ, while accounting for Sex, GM-BHQ, Age, BMI, and experimental venues as control variables. The model fit indices are shown in Table 5.

**Table 5:** Statistics of theorical model 1 and 2.

	CMIN/DF	GFI	AGFI	CFI	AIC	BIC	CAIC
1	4.982	0.966	0.928	0.914	152.627	257.257	281.257
2	4.981	0.966	0.928	0.914	152.605	257.235	281.235

CMIN/DF represents the chi-square value divided by the degrees of freedom. It assesses how well the model fits the data, considering the complexity of the model. A lower ratio indicates a better fit, and usually a value less than 3 has an acceptable fit and a value less than 5 would consider as a reasonable fit^37,38^; GFI (Goodness of Fit Index) compares the fit of the hypothesized model to a model where all variables are uncorrelated. Higher values indicating a better fit. In research, GFI values above 0.95 are often considered as an excellent fit^39,40^; AGFI (Adjusted Goodness of Fit Index) has some similarities to GFI, but adjusts for the number of estimated parameters within the model. Empirically, more than 0.90 is considered as an acceptable fit^41^; CFI (Comparative Fit Index) compares the hypothesized model to a null model with more than 0.90 being an acceptable fit^42^.

Given these statistically standard, it is apparent that all the values of the indicators shown in Table 6 fall within the acceptable range. Furthermore, AIC, BIC and CAIC are information criteria that can be used as model selection tools to compare models fit to the same data. Akaike Information Criterion (AIC)^43^ strikes a balance between lack of fit and the model complexity. Bayesian Information Criterion (BIC)^44^ has imposed a larger penalty on the model complexity as compare to AIC, often favors a sparser model. Consistent AIC (CAIC)^45^ is an extension of AIC, making it asymptotically consistent and imposing stricter penalties for overparameterization. Overall, all three information criteria listed above suggest that a model with smaller values achieves a better balance between lack of fit and model complexity, indicating a superior model.

The sample size is *n* = 578, with significance levels indicated by * *p* < 0.05; ** *p* < 0.01; *** *p* < 0.001. The correlations between variables are included in the figure. Based on the 7 metrics that employed in the table for the comparison between these 2 models, although model 1 and model 2 appeared to have similar values in term of CMIN/DF, GFI, AGFI and CFI, model 2 has a slightly lower values in AIC, BIC, and CAIC. With lower values indicating better model fit. Therefore, model 2 is the best fit.

**Table 6:** Path coefficients for model 1.

Path			Path Coefficient
Fatigue	⇒	FA-BHQ	-0.052*
Curiosity	⇒	Fatigue	-0.090***
Curiosity	⇒	FA-BHQ	0.031*
Age	⇒	FA-BHQ	-0.057***
Sex	⇒	FA-BHQ	-1.110***
GM-BHQ	⇒	FA-BHQ	0.121***
Venue 3	⇒	FA-BHQ	3.620***
Venue 4_1	⇒	FA-BHQ	-1.312*
Venue 1	⇒	FA-BHQ	-3.021***
			**Covariance**
Age	⇔	GM-BHQ	-63.170**
Sex	⇔	GM-BHQ	1.342***
Venue 4_1	⇔	Sex	0.030***
Venue 1	⇔	Curiosity	-0.493***
GM-BHQ	⇔	Venue 4_1	0.240***
Error of Fatigue	⇔	Venue 3	-0.318***

**Table 7:** Path coefficients for model 2.

Path			Path Coefficient
Fatigue	⇒	FA-BHQ	-0.052*
Fatigue	⇒	Curiosity	-0.151**
Curiosity	⇒	FA-BHQ	0.031*
Age	⇒	FA-BHQ	-0.057***
Sex	⇒	FA-BHQ	-1.110***
GM-BHQ	⇒	FA-BHQ	0.121***
Venue 3	⇒	FA-BHQ	3.620***
Venue 4_1	⇒	FA-BHQ	-1.312*
Venue 1	⇒	FA-BHQ	-3.021***
			**Covariance**
Venue 4_1	⇔	GM-BHQ	0.240***
Age	⇔	GM-BHQ	-63.170***
Venue 4_1	⇔	Sex	0.030***
GM-BHQ	⇔	Sex	1.342***
Fatigue	⇔	Venue 3	-0.306***
Error of Curiosity	⇔	Venue 1	-0.518***

As indicated in Tables 6 and 7, while controlling for Sex, GM-BHQ, and experimental venues, FA-BHQ continues to exhibit significant correlations with Curiosity (*b* = 0.031, *p* = 0.042, and Fatigue (*b* = − 0.052, *p* = 0.01) at the 5% level. Given the persistence of these two significant relationships in both model 1 and model 2, it is worthwhile to explore whether mediator effects are present in this relationship.

The mediation analysis, built upon the model 1 and 2 depicted in Figs. 4 and 5, investigated the relationships among Curiosity/Fatigue as independent variables, FA-BHQ as the dependent variable, and Fatigue/Curiosity as the mediator respectively. The analysis encompassed the computation of direct and indirect effects through bootstrap procedures, utilizing 2000 samples, and established bias-corrected bootstrap confidence intervals set at 90%. The outcomes of this analysis are detailed in Table 8.

**Table 8:** Mediation testing with fatigue and curiosity as a mediator.

		Total Effect	Direct Effect	Indirect Effect	Remark
Model 1	Curiosity > Fatigue > FA	0.036*	0.031*	0.005*	Supported
Model 2	Fatigue > Curiosity > FA	-0.057*	-0.052*	-0.005*	Supported

The findings reveal that fatigue serves as a mediator in the relationship between Curiosity and FA-BHQ, indicated by a significant indirect effect (*b* = 0.005, *p* < 0.015). Therefore, Hypothesis 3 are supported. These results suggest that incorporating Fatigue as a mediator amplifying the connection between Curiosity and FA, intensifying the influence of Curiosity on FA levels. Moreover, the results also shows that Curiosity mediates the relationship between Fatigue and FA, as the indirect effects are also statistically significant (*b*=-0.005, *p* = 0.027), intensifying the negative impact of Fatigue on FA-BHQ. Consequently, we can accept hypothesis 4.

**Table 9:** Partial correlation analysis between fatigue/curiosity and components of FA-BHQ while controlling sex, age and GM-BHQ.

	Mean	SD	Fatigue	Curiosity
Corpus Callosum	100.538	4.807	-0.115***☨	0.119***☨
Fornix	100.872	5.265	-0.095*☨	0.138***☨
Internal Capsule	100.452	4.462	-0.095*☨	0.138***☨
Corona Radiata	101.082	5.231	-0.079	0.150***☨
Posterior Thalamic Radiation	100.347	5.536	-0.122***☨	0.072
Sagittal Stratum	101.052	4.876	-0.066	0.066
External Capsule	100.558	4.176	-0.059	0.091*☨
Cingulum	99.382	4.173	-0.080	0.169***☨
Superior Longitudinal Fasciculus	100.295	4.604	-0.056	0.089*☨
Uncinate Fasciculus	98.959	6.453	-0.073	0.058

* *p* < 0.05; ** *p* < 0.01; *** *p* < 0.001.☨: *p* < 0.05 for multiple comparisons using the Benjamini and Hochberg method.

Additionally, as shown in Table 9, individual analysis of FA-BHQ values of the specific brain regions reveal significant correlations with Curiosity and Fatigue. Specifically, the corpus callosum (*r* = -0.115, *p* < 0.001), internal capsule (*r* = -0.095, *p* < 0.05), fornix (*r* =- 0.095, *p* < 0.05) and posterior thalamic radiation (*r* = -0.122, *p* < 0.001) show significant correlation with fatigue. Conversely, the FA-BHQ values of seven brain regions, including the corpus callosum (*r* = 0.119, *p* < 0.001), fornix (*r* = 0.138, *p* < 0.001), internal capsule (*r* = 0.138, *p* < 0.001), corona radiata (*r* = 0.150, *p* < 0.001), external capsule (*r* = 0.091, *p* < 0.05), cingulum (*r* = 0.169, *p* < 0.001), and posterior thalamic radiation (*r* = 0.089, *p* < 0.05), exhibit significant correlation with curiosity individually.

Moreover, in the multiple comparisons using the Benjamini & Hochberg method, the variables Curiosity and Fatigue remained statistically significant at the 5% level, while no additional variables reached significance after correction for multiple comparisons. Consequently, we can infer that the posterior thalamic radiation, corpus callosum, fornix, and internal capsule are closely correlated with fatigue. conversely, the corona radiata, cingulum, fornix, internal capsule, corpus callosum, external capsule, and superior longitudinal fasciculus are closely correlated with Curiosity.

## Discussion

In comparing the two models, Model 2, where curiosity serves as the mediator, emerges as the preferred model. Intriguingly, this model also reveals a significant mediating effect. Since Curiosity and Fatigue are negatively correlated, using Curiosity as the mediator between Fatigue and FA-BHQ enhances the negative impact of Fatigue on FA-BHQ. The findings of this study expand our understanding of the complex relationship between curiosity, fatigue, and brain health. While prior research has extensively focused on fatigue’s detrimental effects on brain health in the way of reducing FA^5^ and increased mean diffusivity in white matter tracts^10^. This study not only proved the negative impact of fatigue towards brain but also uniquely highlights the positive role of curiosity in preserving and enhancing FA-BHQ.

### Reassessing the significance of curiosity

In the face of the challenges posed by an aging population, especially in developed societies, the increasing longevity of individuals has elevated the importance of maintaining brain health in later years. The quest for a healthier brain and, by extension, a healthier life has become a global imperative. Understanding the lifestyle factors that contribute to improved brain health is crucial in addressing the multifaceted aspects of aging and promoting overall well-being. While past literature has extensively explored the connection between fatigue and FA-BHQ, the relationship between curiosity and its potential impact on FA or other indicators of brain health has received comparatively less attention. This research adds to the growing body of evidence of how curiosity may influence the structure of the brain by starting up with correlational research.

This research has revealed compelling evidence pointing to a positive correlation between curiosity and brain structure, as measured by FA-BHQ. This finding suggests that individuals who exhibit higher levels of curiosity tend to have healthier brain structures. Conversely, a negative correlation was observed between FA-BHQ and fatigue, indicating that increased fatigue is associated with less optimal brain health. Notably, there is a greater number of brain regions that are partially positively correlated with Curiosity, while controlling for age, sex, and GM-BHQ. This suggests that, based on the MRI data, curiosity plays a significant role in positively influencing the health of more brain regions while controlling the effect of age, sex and GM-BHQ. The intricate interplay between curiosity, fatigue, and brain health highlights the complexity of the factors influencing cognitive well-being.

Based on the regression results presented in Table 7, a significant negative correlation between curiosity and fatigue is evident (*r* = -0.151, *p* < 0.01). This finding implies that as curiosity increases, the level of fatigue in individuals tends to decrease. Higher curiosity level of an individual appears to be associated with a more active and driven lifestyle, fostering a greater inclination to explore the surrounding world. This finding also aligns with our expectations and is consistent with previous literature. For instance, Shen et al.^46^ discovered that fatigue is likely to reduce the exploration behavior of the individual. Valji et al.’s^11^ study linked curiosity to neural mechanisms involving reward processing and motivational behavior. Moreover, the finding that curiosity mitigates fatigue’s impact on FA-BHQ supports Garrosa et al.’s 2017^12^ research showing curiosity’s protective effect against emotional exhaustion and disengagement.

Traditionally, literature has predominantly emphasized the link between fatigue and brain health, relegating curiosity to a secondary role. However, the findings of this study challenge this prevailing notion. Contrary to expectations, curiosity emerges as a potentially more influential factor concerning FA. This novel implication prompts a paradigm shift in prioritizing the exploration of curiosity’s impact on brain health, offering a fresh perspective for future research endeavors in the domain of neuroscience and cognitive health.

### Implication behind fatigue and brain health

While the negative relationship between fatigue and FA-BHQ is established, the strength of this correlation intensifies when considering fatigue as a mediator in the model. As illustrated in Table 7, the direct effect between curiosity and FA-BHQ is 0.31 with *p* value < 0.05, but it increases to 0.36 when fatigue is included into the model. This result suggests that even when fatigue is considered, the influence of curiosity on maintaining a higher level of FA-BHQ is more pronounced, emphasizing the significant impact of curiosity on brain health.

### Components of FA-BHQ

This study also showed that the FA-BHQ values of the corpus callosum, internal capsule, fornix, posterior thalamic radiation are significantly correlated with fatigue while corpus callosum, fornix, internal capsule, corona radiata, external capsule, cingulum and posterior thalamic radiation are significantly correlated with curiosity given control for sex, age and GM-BHQ.

The potential mechanism happening in the brain function could be considered as follows: Firstly, the corpus callosum serves as the bridge connecting the left and right hemispheres of the brain, facilitating the exchange of information between the two halves. Recent studies suggest that mental fatigue is becoming more commonly associated with the deviated reorganization of functional connectivity among different brain regions^47^; Secondly, the internal capsule (IC) which has a subcortical white matter structure with a location in the inferomedial portion of each cerebral hemisphere^48^. IC is part of limbic-thalamo-cortical circuitry which have been linked to impaired top-down emotion regulation systems in PTSD^49,50^. Additionally, the IC serves as a pathway to the thalamus, which, in turn, has white matter pathways connecting with the amygdala^51,52^. The research conducted by Grillon, et al., in 2015^53^ suggests that engaging in cognitive tasks and experiencing subsequent mental fatigue can compromise the ability to regulate emotions. This result implies that mental fatigue is essential in emotion regulation. Next, posterior thalamic radiations, which is part of Thalamocortical radiations, connect the thalamus to the cerebral cortex. The thalamus serves as the central relay center of the brain, forwarding all sensory information, except for olfaction, to the cerebral cortex for additional processing^54^. Individuals with multiple sclerosis who exhibit high scores in sensory sensitivity also tend to experience greater levels of cognitive fatigue and a lower quality of life^55^. Lastly, fornix, which is the principal axonal tract of the hippocampus, has a connection between the hippocampus and various modulatory subcortical structures. Research indicates that the fornix plays a crucial role in memory formation by serving as a pathway for theta rhythms and acetylcholine^56^. In the meanwhile, research has shown that physical fatigue from a running exercise has negative effect on short-term memory^57^. Building on the aforementioned information, the relationship between FA-BHQ and fatigue becomes evident. When individuals experience physical or mental exhaustion, the brain’s ability to process information, especially in relation to complex tasks and the control of facial muscles, becomes challenging. This finding aligns with well-documented observations in the literature, emphasizing how fatigue adversely affects cognitive processes such as attention, memory, and decision-making.

Moving on to the curiosity side, we observed that there are seven brain parts that are positively correlated with curiosity, namely, corpus callosum, fornix, internal capsule, corona radiata, external capsule, cingulum and posterior thalamic radiation. Firstly, fornix, which contributes mnemonic representations to deep brain structures, is also influencing motivated behaviors^56^. Research has shown that curiosity naturally emerges in tandem with cognition and motivation and serves as a motivator for cognitive processes^58^. Secondly, corona radiata is a part of the limbic thalamo cortical circuits, encompassing thalamic projections from the internal capsule. The pathway connects the thalamus and the amygdala to the cortex^51,52^, specifically involving the prefrontal cortex gray matter areas associated with top-down emotion regulation systems^59^.In this way, the corona radiata has a function in emotional regulation processes^60^. In the research conducted by Leonard, N. H., & Harvey, M^61^. in 2007 has shown a close relationship between absorption curiosity and attention to emotions, clarity of emotions, and repair of emotions which are known as parts of emotional regulation process. Thirdly, the external capsule, situated between the putamen and claustrum, constitutes a gathering of white matter fibers. However, its structural organization and precise functional role are not fully understood at present^62^. Within the external capsule are striatal fibers, facilitating connections such as those between the primary sensorimotor cortex and the putamen, as well as between the supplementary motor area and the caudate nucleus^63^. As a result, it is believed that the external capsule plays a crucial role as a connection between cortical motor regions and the basal ganglia, contributing to the engagement of the basal ganglia in motor control. The basal ganglia participate in various parallel and functionally segregated cortical-subcortical circuits, supporting diverse sensorimotor, cognitive, and emotional-motivational brain functions. Litman^15^, has proposed an integrative I/D/wanting-liking model to prove that the nature of curiosity as an emotional-motivational state. Next, the cingulum bundle is a pathway through which the cingulate cortex sends projections to both the amygdala and prefrontal cortex. This neural pathway extends from the prefrontal cortex to the entire medial collateral limbic complex, including the parahippocampal region^64^. Recent research found that curiosity is triggered by substantial prediction errors that are assessed or evaluated. This cycle allows memory encoding by heightening attention, exploration, and the pursuit of information. Moreover, it strengthens the consolidation of information obtained during a state of curiosity through dopaminergic neuromodulation of the hippocampus which comprises parahippocampal region^65^. Moving to internal capsule, as abovementioned, it is part of limbic-thalamo-cortical circuitry that is associated with impaired top-down emotion regulation systems in PTSD. In the past literature, it has been proven that curiosity has a close relationship with emotional regulation. As during the search process, the individual is likely to experience both pleasure and pain and it is important for the individual to manage those feeling to navigate a pathway through the process^66^. Lastly, the relationship between the corpus callosum and curiosity lacks direct support due to the limited research on the brain mechanisms associated with curiosity. However, previous studies have established a positive correlation between openness and white matter integrity in the splenium of the corpus callosum^67^. Additionally, research by Tan et al.^68^ demonstrated a significant positive correlation between openness and curiosity. Consequently, we can conclude that, under the fMRI level investigation, there is a positive correlation between white matter integrity in the splenium and curiosity, mediated by the trait of openness. This conclusion is consistent with our findings.

### Limitation

The limitations of this research contain four aspects. Firstly, the sample size and participant characteristics, as well as the analytical methods used, pose potential constraints. While our study included data from 578 healthy Japanese participants with an age range of 20 to 66, which is relatively large compared to some studies in this field, but the sample size still limits the generalizability of our findings. To establish the universality of our results, it would be better to include a more diverse participant pool with varying cultural backgrounds. Additionally, the use of cross-sectional data in this research prevents us from establishing causal relationships and understanding how the relationships between variables may change over time. Future research should aim to resolve these limitations by expanding the participant pool and employing diverse analytical approaches to further explore and confirm the identified relationships.

Secondly, the limitation of selection of the control variables used in the research might reduce the reproducibility of the results of this study. While we incorporated sex, age, GM-BHQ, and experiment venues as control variables, the exclusion of factors like income level, marital status, and knowledge level could potentially affect the robustness and social implications of our findings. Future research should consider expanding the set of control variables to enhance the comprehensiveness and applicability of the study.

Thirdly, the insufficient evidence in past literature hinders the validation of our findings. In our research we found corpus callosum is positively related with curiosity with statistical significance. However, as mentioned above, direct support for this finding in existing literature is lacking. Although we derive a conclusion through mediating factor of openness, the relationship between curiosity and corpus callosum as well as the brain mechanism on curiosity still worth exploration in future studies. This exploration is essential to substantiate and enhance the understanding of our observed results.

Next, the absence of D-type curiosity in our research framework limits the scope of our investigation into the multifaceted nature of curiosity. D-type curiosity, as described by Litman^15,16^, represents a drive to resolve specific informational gaps, often accompanied by a sense of deprivation or urgency. While our findings offer valuable insights into the relationship between curiosity and brain health, they are confined to the enjoyment-driven, exploratory aspects of curiosity measured by the CEI-II. This limitation restricts our ability to examine the broader dimensions of curiosity, particularly the unique ways D-type curiosity might impact brain function, cognitive persistence, or the resolution of uncertainty. Future research should incorporate measures specifically designed to assess D-type curiosity, such as the Curiosity as a Feeling-of-Deprivation (CFD) scales, to provide a more comprehensive understanding of how both dimensions of curiosity influence brain health and cognitive processes.

Lastly, it worths to highlight that the outcome of this study was based on a classical approach for mediation analysis. There are several limitations on the classic approach such as ignoring the confounding factor between mediator and outcome variables. Therefore, in future work, the researchers should adopt Causal Mediation Analysis or Counterfactual-based Mediation Analysis to further validate and refine these findings.

## Conclusion

In this research encompassing 578 participants in Japan, several findings have emerged. Firstly, there exists a positive correlation between brain health, as assessed by FA, and Curiosity measured through CEI-II questionnaires. Secondly, a negative correlation was established between FA and Fatigue, gauged by CFS questionnaires. Moreover, a mediating effect of fatigue was identified in the relationship between curiosity and FA and another mediating effect of curiosity between fatigue and FA is observed. Notably, it was found that, in contrast with fatigue, which has been heavily studied in the neuroscience field, curiosity has significant relationship with more FA regions. This raises the attention on the brain mechanism associated with curiosity and its potential implications to improve brain health.

## Data Availability

The datasets generated during the current study are not publicly available but are available from the corresponding author upon reasonable request.
